# Supplementary material for: Revisiting the Role of the Leucine Plug/Valve in the Human ABCG2 Multidrug Transporter
Source: Int J Mol Sci. 2025 Apr 24;26(9):4010. doi: 10.3390/ijms26094010 (PMC12071886; doi:10.3390/ijms26094010)

Supplementary material

Revisiting the role of the leucine plug/valve in the human ABCG2 multidrug transporter

Supplementary Figure S1. Influence of cholesterol on the ATPase activity of ABCG2 variants expressed in Sf9 membrane vesicles. Effect of **(A)** quercetin (QUE) (1.6 M) and **(B)** prazosin (PRAZ) (20 M) on the ATPase activity on ABCG2 variants.

Stimulation of the ATPase activity of the wild-type (wt) and mutant ABCG2 was measured in the absence (noC) and presence of cholesterol (applied as 2 mg/ml cholesterol-cyclodextrin - Chol-CD complex). In the control experiment cholesterol-free cyclodextrin (CD) was used. It should be mentioned, that unloaded cyclodextrin may further decrease membrane cholesterol content in the Sf9 membranes. * p<0.01 basal and drug treatment pairs were compared

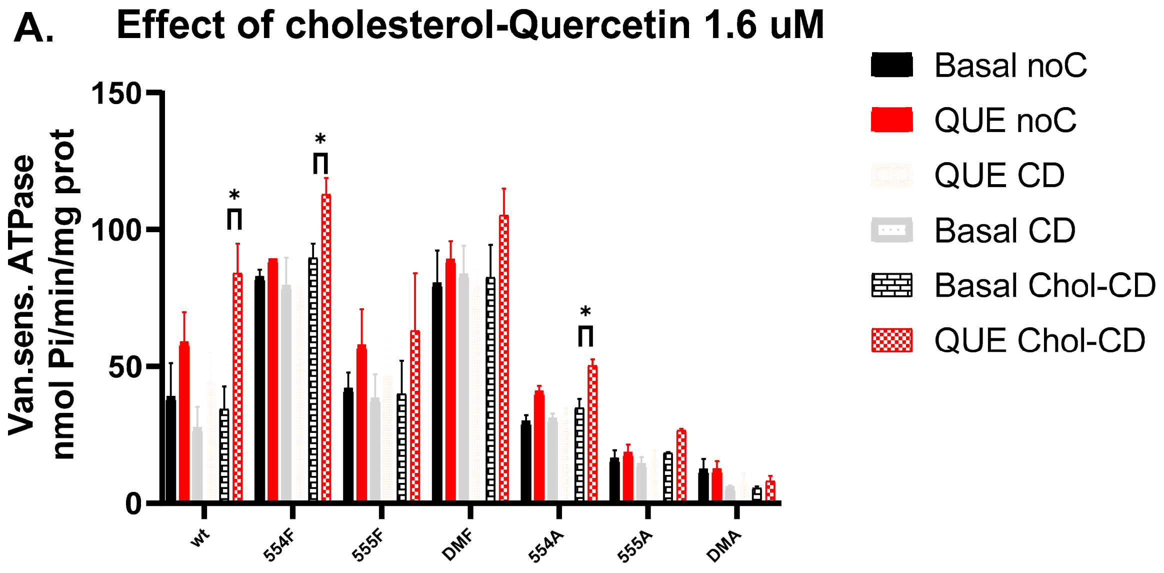

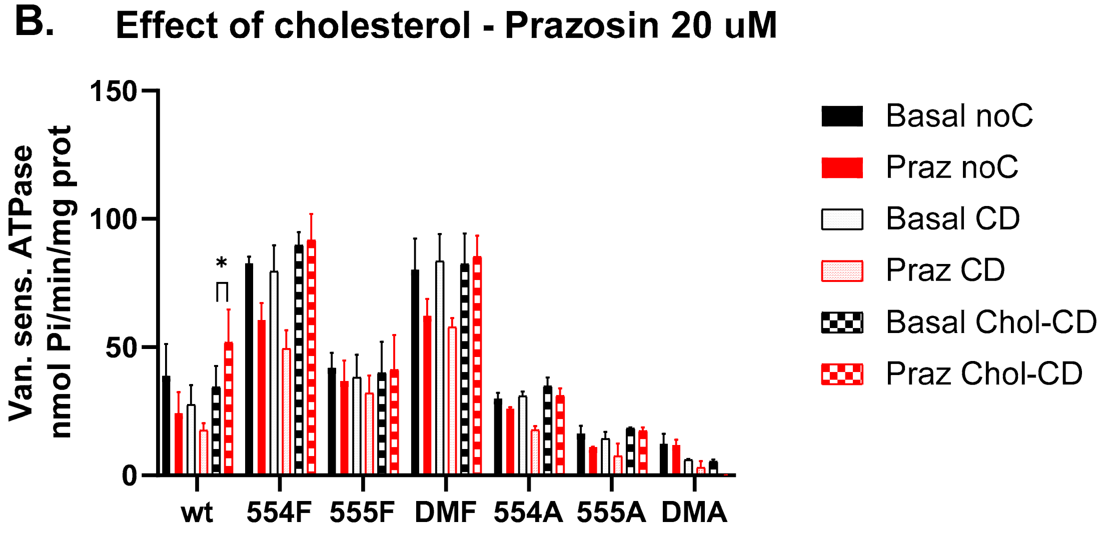

Supplementary Figure S2. Interaction of various substrates with ABCG2 variants measured by the ATPase assay in Sf9 cell membranes.

In a previous paper (DOI 10.1124/dmd.113.055731) we have published that the bile acid-like detergent CHAPS is a modifier of ABCG2 protein that is able to reduce the basal ATPase activity without altering the substrate stimulation of it. We can use CHAPS, as a sensitizer of the assay to see better the substrate effects.

Supplementary Figure S2 shows the relative ATPase activity changes compared to the basal activity. CHAPS (4 mM) was able to reduce the basal activity of L555F similarly to the wt, and a little less that of the L554F. The basal activity of L554A and DMF (the high basal activity variants) were insensitive to CHAPS.

Three activator substrates (quercetin 1.6, prazosin M, nilotinib 1 M) and one inhibitory substrate (elacridar M) were investigated in this setup. We have found that the results for L555F were similar to the wt ABCG2. L554F and DMF were less sensitive to all drugs. L554A results were more unexpected – while CHAPS was not able to decrease the basal activity it could increase slightly the stimulatory effect of strong substrates (quercetin and nilotinib). Currently we have no explanation for this phenomenon, but these data point to a structural alteration by this mutation as well (In silico modelling the ABCG2 interaction with cholesterol and bile acid is not available, it can be an allosteric site as well.) * p<0.01 compared to the baseline

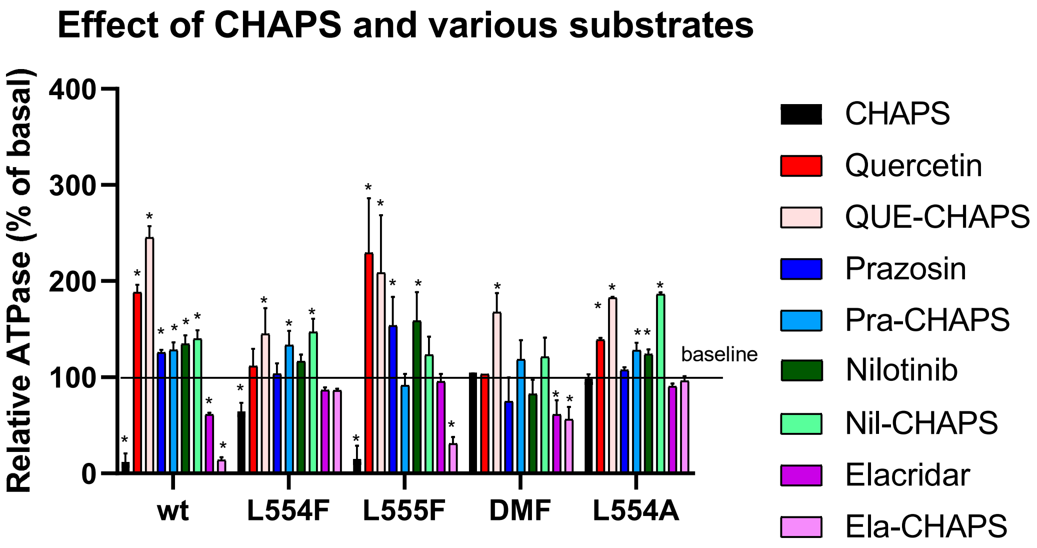

Supplementary Figure S3. Dynamics of ABCG2 variants determined in equilibrium MD simulations. (A) ABCG2 structure (PDBID:6hij) was colored by cyan (chain A) and pale cyan (chain B). The central four helices (TH2, TH5, TH2′ and TH5′) are highlighted with L554 (purple) and L555 (red) valve residues. **(B)** The ABCG2 proteins were stable in MD simulations. RMSD (Root Mean Square Deviation) plots, calculated relative to the initial structure, show values not exceeding 5–6 Å, indicating the stability of a protein of this size during the simulations (n = 3 for each system). **(C)** No notable differences in dynamics were observed among the ABCG2 variants. RMSF (Root Mean Square Fluctuation) values were calculated using conformations from the final 250 ns of the simulations. These values represent the positional fluctuations of each Cα atom around its average position. GROMACS tools also convert RMSF values into pseudo B-factors, allowing numerical comparison with B-factors derived from X-ray crystallography. These pseudo B-factors are embedded in the PDB files, allowing the structures to be visualized using colored wire representations, where regions of higher flexibility appear as thicker wires in warmer colors.

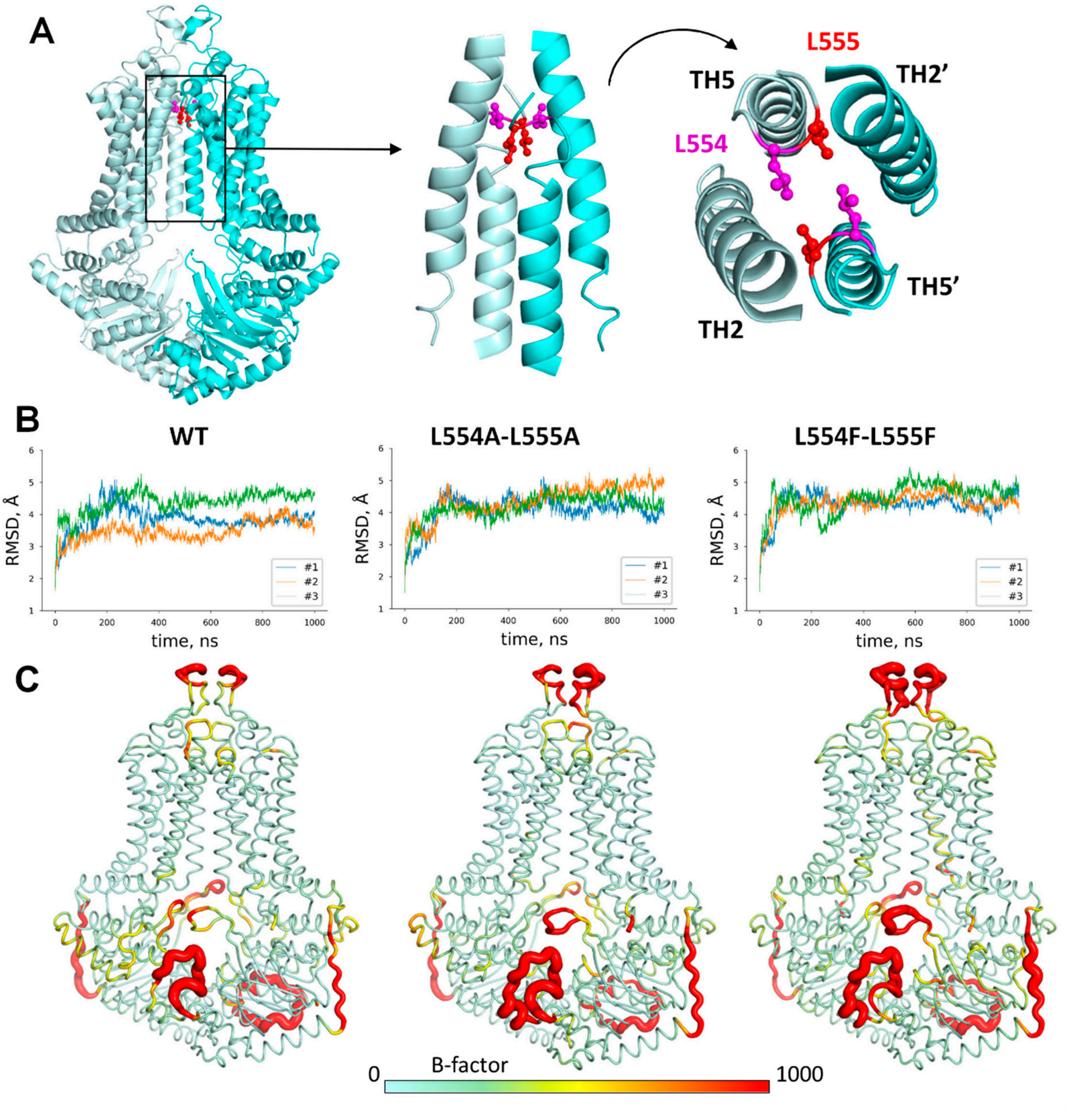

Supplementary Figure S4. Altered contacts of the TM2 and TM5 helices. Contacts between TM2/TM5 from the same protomer (chain A/chain A) (A, C, E) and TM2/TM5 from the opposite protomers (chain B/chain A) (B, D, F) were calculated between Cα atoms at a 7 Å cutoff. This figure shows the same results as the main text Figure 5 but focusing on the opposite TM5 (chain A).

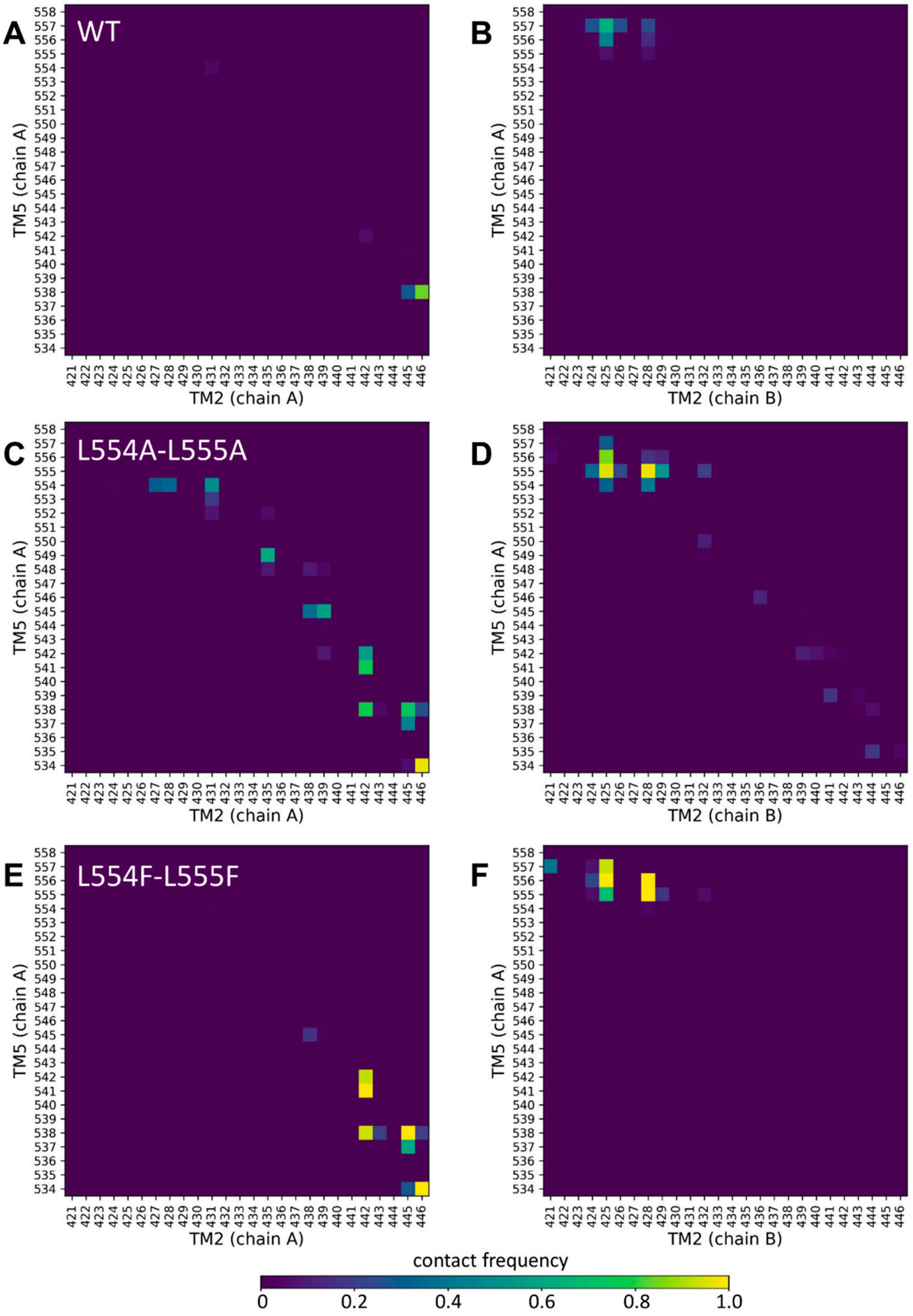

Supplement: Supplementary file 1 [file ijms-26-04010-s001.zip › ijms-3557099-supplementary.pdf]
